# Management of Immune-Related Adverse Events from Immune-Checkpoint Inhibitors in Advanced or Metastatic Renal Cell Carcinoma

**DOI:** 10.3390/cancers14184369

**Published:** 2022-09-08

**Authors:** Katharina Leucht, Nalyan Ali, Susan Foller, Marc-Oliver Grimm

**Affiliations:** Department of Urology, University Hospital Jena, 07747 Jena, Germany

**Keywords:** immune-related adverse events, side effects, immune-checkpoint inhibitors, renal cell carcinoma, immune therapy, adverse reactions

## Abstract

**Simple Summary:**

Today, most patients with metastatic renal cancer receive systemic treatment with so-called immune-checkpoint inhibitors that shall activate a patient’s immune system. For patients without prior therapy, these therapeutic agents are combined with a second immunotherapeutic drug or with a therapeutic agent intended to reduce the tumour’s blood supply, namely tyrosine kinase inhibitors directed against the vascular endothelial growth factor receptor (VEGFR-TKI). Both parts of the combination therapy cause side effects that need to be treated and handled differently depending on the therapeutic agent responsible for the complaints. Therefore, it is of crucial importance to recognize the side effects and relate them to the right therapeutic agent. Within this review we describe the most frequent immune-related side effects of immune-checkpoint inhibitors, especially focusing on their distinction from side effects caused by VEGFR-TKI. Additionally, we explain the management of these complaints as well as their impact on the therapy.

**Abstract:**

Immune checkpoint inhibitors (ICI) are now, among other cancers, routinely used for the treatment of advanced or metastatic renal cell carcinoma (mRCC). In mRCC various combinations of ICIs and inhibitors of the vascular epidermal growth factor receptor tyrosine kinase (VEGFR-TKIs) as well as dual checkpoint inhibition (nivolumab + ipilimumab), the latter for patients with intermediate and poor risk according to IMDC only (international metastatic renal cell carcinoma database consortium), are now standard of care in the first line setting. Therefore, a profound understanding of immune-related adverse events (irAE) and the differential diagnosis of adverse reactions caused by other therapeutic agents in combination therapies is of paramount importance. Here we describe prevention, early diagnosis and clinical management of the most relevant irAE derived from ICI treatment focusing on the new VEGFR-TKI/ICI combinations.

## 1. Introduction

Immune checkpoint inhibitors (ICI) have been established as therapies for a growing number of cancer types. These antibodies target programmed cell death protein 1 (PD-1), its ligand (PD-L1), or cytotoxic T-lymphocyte-associated protein 4 (CTLA-4) [1]. Originally used as monotherapy, ICI are now frequently combined with tyrosine kinase inhibitors directed against the vascular endothelial growth factor receptor (VEGFR-TKI) or other immunotherapies (dual checkpoint inhibition: anti-PD-(L)1 plus anti-CTLA-4) [2].

ICI are associated with the occurrence of immune-related adverse events (irAE) which markedly differ from those of VEGFR-TKI [1,3,4], but may cause similar symptoms. Delays in recognition and treatment of irAEs may lead to exacerbation of symptoms and further complications [1].

ICI are used in therapeutic indications often managed by multidisciplinary teams. With regard to genitourinary cancer, ICIs are best established in metastatic renal cell carcinoma (mRCC) and are currently analysed in clinical trials in the (neo-)adjuvant setting [2]. Hence, a thorough understanding of the basics, indications and specific features of ICI is extremely important and will be the subject of this article in terms of diagnosis and clinical management of the most relevant toxicities.

## 2. Current Therapeutic Situation in mRCC with Respect to Immune Checkpoint Inhibitors

**Figure 1:**
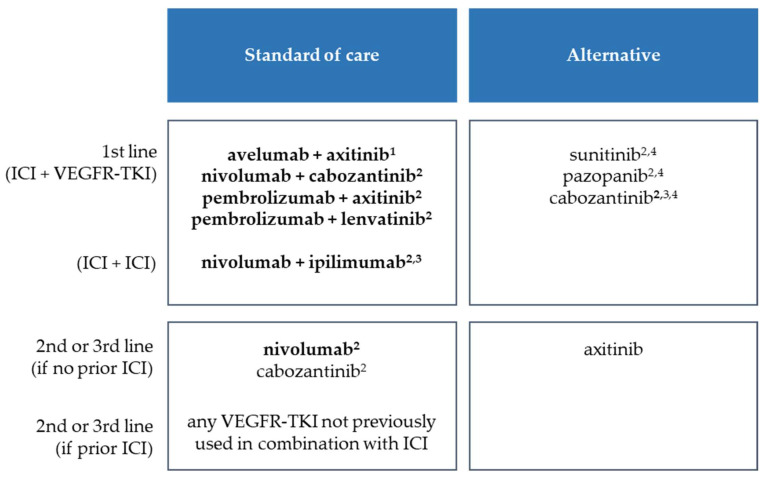
Currently approved and recommended therapies in advanced or metastatic renal cell carcinoma. Therapies with immune checkpoint inhibitor (ICI) component are highlighted in bold. According to EAU guideline Renal Cell Carcinoma [2]: ^1^ approved, but no recommendation by EAU, ^2^ strong recommendation, ^3^ for intermediate and poor risk only, ^4^ in patients with contraindications for ICI.

In first-line mRCC, several ICI/VEGFR-TKI combinations are approved: avelumab+axitinib, nivolumab+cabozantinib, pembrolizumab+axitinib, and pembrolizumab+lenvatinib (alphabetical order, Figure 1). Furthermore, dual checkpoint inhibition with nivolumab+ipilimumab is applicable for patients with intermediate and poor risk according to IMDC. If ICI are not used in the first line, nivolumab monotherapy may be used after prior therapy [2].

In first-line mRCC, ICI/VEGFR-TKI combinations demonstrated benefits compared to previous standard sunitinib, a VEGFR-TKI administered as monotherapy. In the pivotal trials, OS improvement has been reported for both ICI/VEGFR-TKI combinations (exception: avelumab+axitinib) and for dual checkpoint inhibition with nivolumab+ipilimumab [5,6,7,8,9]. The benefits of therapy must be individually weighed against the frequency and severity of irAE.

## 3. Frequencies of Treatment- and Immune-Related Adverse Events

The existing data on the incidences of adverse reactions, as found in the Summaries of Product Characteristics (SmPCs) of the European Medicines Agency (EMA) and the respective product informations, derive from pivotal studies. While treating patients in the scope of these clinical trials, experienced investigators evaluate and classify adverse events (AEs) to the best of their knowledge and differentiate, whether there is a reasonable causal relationship between study drug administration and the AE (evidence to suggest a causal relationship, treatment-related AE, trAE) and, especially in the case of ICI, whether an AE might be immune-related (irAE) or not. Adverse reaction frequencies presented in SmPCs may not be fully attributable to a respective drug alone but may contain contributions from the underlying disease or from other medicinal products used in a combination. Hence, stated numbers may overestimate the frequency of occurrence of an adverse reaction with respect to the particular drug.

Based on their frequency, adverse reactions (including irAEs and non-irAEs) are classified as “very common” (≥10%), “common” (1–10%), “uncommon” (0.1–1%), “rare” (0.01–0.1%) and “very rare” (<0.01%). This classification is also used in the present review.

In the metastatic setting, tumour symptoms often dominate patients’ complaints. When combining ICI with VEGFR-TKI the latter very frequently causes more or less chronic adverse events (non-irAE) that can be mitigated by prophylactic and/or therapeutic measures. Due to this “background”, it is even more challenging to identify the relatively rare irAE.

**Figure 2:**
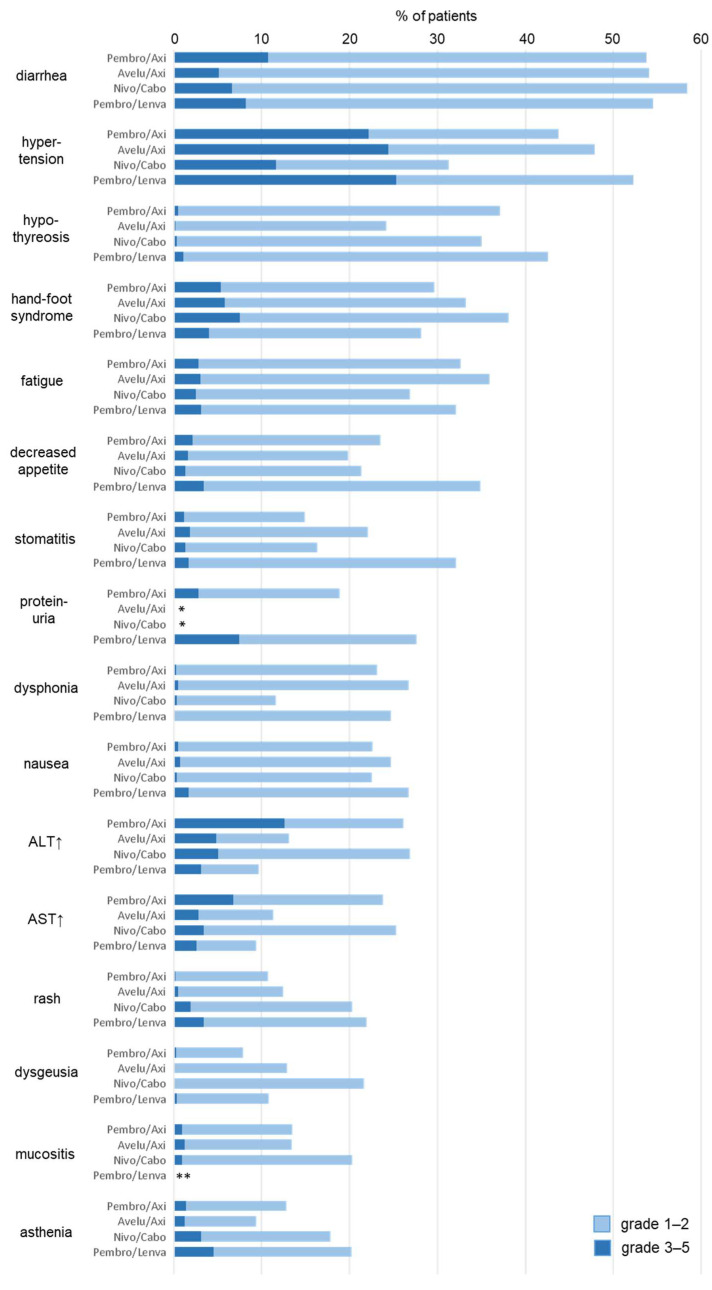
Frequencies of treatment-associated adverse events (trAE) of ICI/VEGFR-TKI combinations in metastatic renal cell carcinoma in ≥20% of patients according to KEYNOTE 426 (pembrolizumab+axitinib, median follow-up: 30.6 months) [12], JAVELIN renal 101 (avelumab+axitinib, median follow-up: 11.6 months) [11], CheckMate 9ER (nivolumab+cabozantinib, median follow-up: 23.5 months) [13], and CLEAR (pembrolizumab + lenvatinib, median follow-up: 26.6 months) [8]. * Not stated, <10% based on trAE of all CTCAE grades; ** <20% based on trAE of all CTCAE grades.

The incidences of “very common” and “common” adverse reactions to the ICI/VEGFR-TKI combinations are summarized in Figure 2. While with ICI most irAEs occur during the first months of treatment please note, however, that non-irAE adverse reactions occur in a time-dependent manner causing higher event rates with longer study follow-up.

If combining an ICI with the VEGFR-TKIs axitinib, cabozantinib or lenvatinib, ICI-associated irAE and “chronic” VEGFR-TKI toxicities appear to add up without supra-additive effects. The VEGFR-TKI-associated adverse reactions mainly consist of diarrhoea, hypertension, fatigue, hypothyroidism, palmar–plantar erythrodysesthesia syndrome (hand–foot syndrome), and gastrointestinal symptoms (nausea, loss of appetite, stomatitis) [10].

The overall incidence of trAE for ICI/VEGFR-TKI combinations in mRCC is reported to be 95–97% for all grades and 57–72% for grade ≥ 3 toxicities [8,11,12,13]. Using dual checkpoint inhibition with nivolumab+ipilimumab in mRCC patients, all grade and grade 3–4 trAE occurred in 94% and 48%, respectively [5].

Considering AEs designated to be immune-related, the whole grade and grade 3–4 incidence for ICI/VEGFR-TKI combinations in mRCC was 38–61% and 9–15%, respectively [8,11,12,13]. With nivolumab+ipilimumab, 81% of patients experienced AEs (all grades) considered to be immune-related (“select AE”), predominantly grade 1–2 [14]. Overall, a comparison of dual checkpoint inhibition-associated AEs with those of ICI/VEGFR-TKI combinations is hardly possible: While with nivolumab+ipilimumab irAE occur temporarily and especially during the first 12 weeks, ICI/VEGFR-TKI toxicity is mainly determined by the chronic nature of adverse reactions caused by VEGFR-TKI.

## 4. Early Diagnosis, Differential Diagnosis and Management of Immune-Related Adverse Events

The organs most frequently affected by ICI toxicity are the skin, liver, colon, lung and endocrine systems. Diagnostically, among common irAE one may distinguish between those primarily becoming apparent via symptoms and those being detected due to laboratory abnormalities. In order to detect primarily symptomatic irAE as early as possible, patients should be asked for rash, pruritus, diarrhoea (colitis), and respiratory symptoms incl. dyspnoea (pneumonitis) on a regular basis. Routine laboratory analysis should include liver values and pancreatic enzymes (amylase, lipase), TSH, and creatinine. In addition to these laboratory parameters blood count, electrolytes, urea, glucose, and, in the case of TKI combinations, differential blood count, albumin, and phosphate should be determined before the start of therapy as well as before each infusion or every 2–3 weeks, respectively (tabular overview in Heinzerling et al. [15]). In case of a non-specific deterioration of a patient’s health status, adrenal or pituitary insufficiency should always be considered and measurement of ACTH, cortisol, prolactin, LH, FSH and estradiol or testosterone is mandatory.

Some irAE are mainly diagnosed by exclusion. This especially applies to:

- Diarrhoea/colitis: stool culture for exclusion of pathogenic agents.
- Hepatitis: liver ultrasound in case of increased transaminases (cholestasis, progression of liver metastasis?), hepatitis serology, testing for potentially hepatotoxic drugs/nutritional supplements.

An additional issue for combination therapies in mRCC is the overlapping toxicity profiles of ICI and VEGFR-TKI. In practice, if an adverse reaction may not be assigned to either the ICI or the VEGRF-TKI component, it is recommended to discontinue the VEGFR-TKI and delay the ICI infusion. An improvement in adverse reactions caused by VEGFR-TKI can be expected, among others, depending on its plasma half-life (axitinib 2.5–6.1 h; cabozantinib 110 h, lenvatinib 28 h; according to the respective EMA SmPCs). Eventually, the type and severity of the adverse reaction as well as the patient’s general condition have to be considered when deciding for or against an early start of corticosteroid therapy to prevent worsening of a potential irAE. If symptoms rapidly subside upon corticosteroids, the adverse reaction most likely, but not evidentially, was immune-related. The early use of corticosteroids must always be carefully considered with regard to possible differential diagnoses since the erroneous classification of an adverse reaction as immune-related can possibly lead to non-essential discontinuation of ICI therapy (see below).

**Figure 3:**
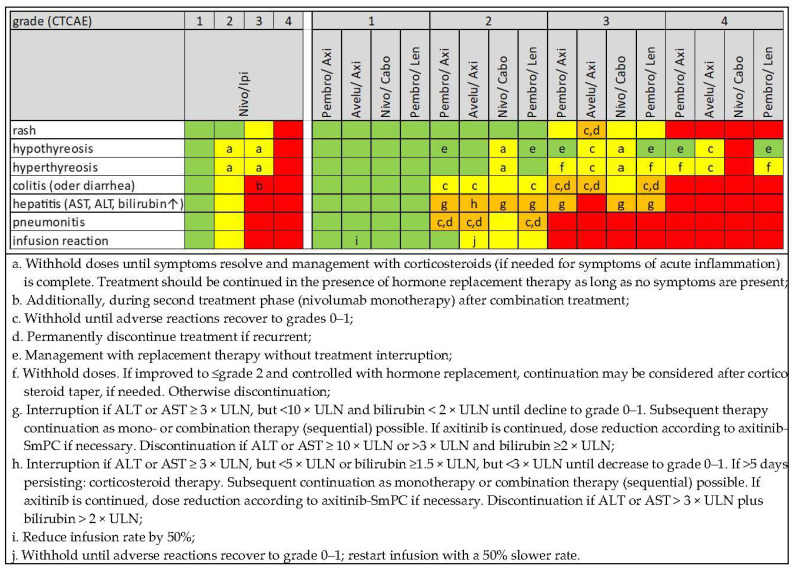
Recommendations for therapy management of immune checkpoint inhibitors (ICI) in ICI-based combination therapy depending on type and severity of important irAE. *Green:* continuation, *yellow:* interruption, *orange:* interruption or discontinuation under certain conditions, *red:* permanent discontinuation. Assessment based on the respective Summaries of Product Characteristics (SmPC) of the European Medicines Agency (EMA) (accessed 22 July 2022). (ir)AE, (immune-related) adverse event; ALT, alanine aminotransferase; AST, aspartate aminotransferase; Avelu, avelumab; Axi, axitinib; Ipi, ipilimumab; Lenva, lenvatinib; Nivo, nivolumab; Pembro, pembrolizumab; ULN, upper limit of normal range; ↑, increased.

For the management of irAE, ICI therapy may be interrupted or permanently discontinued (no dose reductions). In general, despite an irAE of grade 1 according to CTCAE therapy can usually be continued; for grade 2, therapy delay plus corticosteroid administration is suggested; and for grade 4, permanent discontinuation of therapy with concomitant administration of systemic corticosteroids is recommended [16]. Recommendations for the management of grade 3 irAE differ: depending on the affected organ system and the ICI or ICI combination treatment delays or discontinuation are advised (for dual checkpoint inhibition and ICI/VEGFR-TKI combinations summarized in Figure 3).

When ICI/VEGFR-TKI combinations are administered, the VEGFR-TKI dose may be reduced for the management of TKI-associated adverse reactions, regardless of an interruption of the ICI therapy. For axitinib, the dosage can also be gradually increased if well tolerated. It must be noted that the recommended daily starting doses for cabozantinib and lenvatinib differ from those for monotherapy: cabozantinib: 40 mg instead of 60 mg, lenvatinib: 20 mg instead of 18 mg (if combined with everolimus) or 24 mg (monotherapy). Dose re-escalation is possible for axitinib, but not recommended for cabozantinib and lenvatinib [8,11,17,18]. Dose adjustment of VEGFR/TKI according to the respective SmPC may also be required in case of an existing (unavoidable) co-medication with potent CYP3A4/5 inhibitors (and inducers) [19].

When VEGFR-TKI/ICI combinations were administered in clinical studies, high-dose corticosteroids had to be applied as follows: avelumab+axitinib: 11%, nivolumab+cabozantinib: 19%, pembrolizumab+axitinib: 27%, pembrolizumab+lenvatinib: 15% [11,17,18,20]. Upon nivolumab+ipilimumab 29% of patients received high-dose corticosteroids [15].

After irAE symptoms improved to grade 1 corticosteroids should be tapered over 4 weeks. If the corticosteroid dose could be reduced to physiological levels (≤10 mg prednisolone or equivalent) therapy may be resumed. Eventually, after an (ir)AE-associated treatment interruption, it is recommended to sequentially restart therapy, with the VEGFR-TKI being applied first. This may reveal if, against expectations, the observed adverse reaction was caused by the VEGFR-TKI (and therefore probably not immune-related).

Below, frequent irAE are discussed in more detail focusing on ICI/VEGFR-TKI combinations in the mRCC setting. For common adverse reactions, consensus recommendations provide guidance on the clinical management of fatigue [21], diarrhoea [22], nausea and vomiting [23], infusion-related reactions [24], pain [25], or cardiotoxicity [26]. More comprehensive recommendations for all irAE are provided in clinical practice guidelines of the European Society for Medical Oncology (ESMO) [27] and the American Society of Clinical Oncology (ASCO) [28].

## 5. Common irAE per Entity

### 5.1. Skin and Mucosal Toxicity

Pruritus and rash, the clinically most relevant “very common” dermatological adverse reactions, are reversible and well manageable. VEGFR-TKI-associated palmar–plantar erythrodysesthesia (“hand-foot”) syndrome is also “very common” in respective combinations. Prophylactically, skin irritation on hands and feet should be avoided (e.g., by wearing comfortable footwear) and lipophilic urea creams should be applied. The latter may also prevent “common” erythema, dry skin, urticaria, eczema and dermatitis. Grade 1 immune-related skin reactions are treated with moisturizing creams or lotions, newer generation oral antihistamines, or mild topical corticosteroids; ICI therapy may be continued. However, skin-related events of other aetiologies (e.g., infection, vasculitis, contact dermatitis) should be ruled out by follow-up examinations. For grade 2 irAE, additional moderate to strong topical corticosteroids are indicated whereas, for grade 3, strong topical or systemic corticosteroids (0.5–1 mg/kg) are required. In case of grade 3 and 4 symptoms, therapy should be interrupted and discontinued, respectively. If severe skin reactions occur (Stevens–Johnson syndrome, toxic epidermal necrolysis) 1–2 mg/kg (methyl-) prednisolone i.v. should be applied [27].

### 5.2. Hepatobiliary Toxicities

Depending on the ICI administered (as monotherapy or combined) elevated laboratory values for transaminases, alkaline phosphatase, gamma-glutamyltransferase and total bilirubin occur “frequently” to “very frequently”. All may indicate “occasional” to “frequent” hepatitis.

Since immune-related hepatitis usually begins asymptomatically, serum transaminases and bilirubin should be determined before each therapy cycle for early detection. Immune-related hepatitis is primarily associated with increased transaminases as a “transaminitis” while a concomitant increase in bilirubin may indicate another cause. An ultrasound examination of the liver (cholestasis? progression of liver metastases?) and hepatitis serology are recommended for diagnosis by exclusion. In addition, the patient’s medication should be reviewed regarding hepatotoxic drugs. VEGFR-TKI may also cause hepatotoxicity with elevated transaminases which should be taken into account for ICI/VEGFR-TKI combinations. Therefore, the VEGFR-TKI should be primarily discontinued with the laboratory values being closely monitored. The ICI (infusion) therapy may be continued despite the occurrence of grade 1 “transaminitis”, however, this should be weighed thoroughly against a dose delay which may allow for classifying this event as an irAE. In the respective SmPCs, the recommendations for therapy management depend on levels of transaminases and/or bilirubin that may not necessarily correspond to a clear CTCAE grade. In any case, an ICI treatment delay is required for grade 2 laboratory abnormalities [29] (see explanations and recommendations in Figure 3); in certain cases also discontinuation may be required. If transaminases and/or bilirubin remain elevated or continue to rise after several days of VEGFR-TKI treatment discontinuation (cabozantinib > 5–7 days, due to the long half-life), treatment with (methyl-)prednisolone should be initiated. For grade 3, interruption or permanent discontinuation may be required, depending on the ICI/VEGFR-TKI combination applied and on the levels of the respective lab values. Grade 4 changes, however, require permanent discontinuation of treatment and initiation of (methyl-) prednisolone therapy.

If an irAE is suspected and blood values do not improve after 2–3 days, additional mycophenolate mofetil treatment (2 × 1000 mg/day) should be considered and a hepatologist should be consulted. As a third-line therapy with, however, very limited data available, anti-thymocyte globulin or tacrolimus may be used. Provided an adequate treatment, hepatitis improves within 4–6 weeks. If this is not the case, the causal relationship or other co-factors must be re-considered [27].

### 5.3. Gastrointestinal Toxicities

“Very common” ICI-related gastrointestinal toxicities include diarrhoea, nausea, vomiting, constipation, and abdominal pain, as well as increases in lipase and amylase.

Diarrhoea (all grades) is observed in more than 50% of patients undergoing ICI/VEGFR-TKI combination therapy, most frequently caused by the VEGRF-TKI. In contrast to immune-related diarrhoea or colitis, which are usually characterised by an acute onset of pronounced symptoms, TKI-associated diarrhoea usually has an insidious onset. In addition to prophylactic measures such as the intake of frequent small meals and a bland diet, treatment with antidiarrheal drugs may be necessary and compensation for electrolyte or fluid losses should be considered.

Especially in cases of acute or pronounced diarrhoea and/or abdominal discomfort or pain, irAE should be ruled out or, if in doubt, assumed and treated. Enterocolitis may result in anaemia, elevated C-reactive protein (CRP), and decreased serum albumin [30]. In order to rule out causative infection, a stool culture should be examined for bacterial pathogens and clostridioides difficile toxins. If diarrhoea reaches grades 2–3, therapy should be delayed. In case of recurrent or persistent symptoms of grade 3 and for grade 4 events permanent discontinuation is indicated. For nivolumab+ipilimumab therapy should already be discontinued at grade 3. This is due to the significantly increased incidence of (severe) diarrhoea with anti-CTLA-4 therapy (ipilimumab) as compared with anti-PD-(L)1-ICI, as well as its earlier onset in time [31]. Systemic corticosteroids (1–2 mg/kg/day, i.v.) should be considered from grade 2 onwards depending on the severity of symptoms (diarrhoea, abdominal pain, deterioration of general condition) and if symptoms persist despite treatment delay, at the latest in the case of deterioration to grade 3. If there is no response after 3–5 days, infliximab can be added [27]. Due to the risk of recurrence of immune-related colitis, slow tapering of corticosteroids over 4 weeks starting from improvement to grade 1 is strongly recommended.

An increase in amylase and lipase may indicate immune-related pancreatitis. However, frequently these laboratory changes are not accompanied by clinical symptoms (e.g., abdominal pain, vomiting) and therapy can be continued despite grade 3, or possibly even grade 4, with close monitoring. In case of doubt or if the laboratory values continue to rise, it is recommended to interrupt the therapy and sequentially resume it after improvement. Regardless of this, clear manufacturer recommendations for pancreatitis exist for atezolizumab and avelumab+axitinib: For atezolizumab, therapy interruption is recommended in cases of confirmed grade 2–3 pancreatitis, and therapy discontinuation is recommended in cases of grade 4 or repeated occurrence of grade 2–3. In contrast, for avelumab+axitinib, regardless of CTCAE grade, therapy is interrupted if pancreatitis is suspected and discontinued if the diagnosis is confirmed.

### 5.4. Endocrinological and Metabolic Toxicity

The onset of immune-related endocrinopathies is slow. Additionally, their resolution can take several weeks and these irAE are—in contrast to most others—frequently not reversible. Appropriate patient education including information on the possible need for long-term hormone replacement therapy is recommended.

Among the “common” to “very common” ICI-related immunoendocrinopathies are diseases of the thyroid gland (hypothyroidism, less frequently hyperthyroidism or thyroiditis). Their high incidence with nonspecific symptoms necessitates close monitoring of TSH (also fT3, and fT4 if TSH is repeatedly elevated or depressed). If hypothyroidism has been diagnosed, thyroid hormone substitution (L-thyroxine, initial dose 50 µg) should be initiated depending on clinical symptoms. Mild and asymptomatic hyperthyroidism may be initially observed and convert to hypothyroidism as it progresses. In symptomatic patients, beta-blockers might be useful and, in case of doubt, thyroid sonography and/or determination of thyroid autoantibodies (MAK, TAK, TRAK) may be useful (differential diagnosis: thyroiditis, Basedow’s disease) [27]. Delay of ICI therapy until improvement of symptoms is recommended in CTCAE grade 3 according to the SmPC. Treatment may frequently be resumed after initiation of a hormone replacement therapy.

Immune-related adrenal insufficiency or hypophysitis occurs “occasionally” with ICI monotherapy and “frequently” with combination therapies. Adrenal insufficiency may manifest with various nonspecific symptoms (signs of dehydration, hyperkalemia, hyponatremia, hypotension, dizziness, possibly shock); occasionally, acute adrenal insufficiency may occur [32]. Whether the adrenal insufficiency is secondary to (sometimes partial) pituitary insufficiency, is determined by the constellation of laboratory values (see below).

Immune-related hypophysitis can lead to local swelling and hormonal dysfunction, most commonly presenting as central adrenal insufficiency (see above). Therefore, hypophysitis is diagnosed by the detection of decreased ACTH, LH, FSH, TSH, and prolactin, and corresponding decreased cortisol and estradiol/testosterone. Magnetic resonance imaging (MRI) of the brain may confirm an enlarged pituitary gland. If cortisol is decreased and ACTH is increased, primary adrenal insufficiency is present.

Symptoms of pituitary inflammation are nonspecific and include those of adrenal insufficiency (see above) as well as headache, visual disturbances, and dizziness. In moderate symptoms (grade 2) of adrenal insufficiency or hypophysitis, corticosteroid replacement may be sufficient; in more severe cases (grades 3–4), initial high-dose steroids are required to treat the “-itis” or central symptoms.

Immune-related endocrine adverse reactions are often accompanied by irreversible destruction of the glands, thus leading to insufficiency. Then, a permanent substitution of thyroid hormones or cortisone becomes necessary. For the latter, if tapering of corticosteroids has been attempted without success, the use of hydrocortisone is recommended to avoid the additional use of the mineral corticosteroid substitute fludrocortisone [33,34].

For type I diabetes mellitus as an “occasional” irAE it is recommended to regularly monitor blood glucose levels, especially in cases of polydipsia or -uria. In severe cases ketoacidosis is possible [35] and should be treated according to established guidelines. Insulin substitution and treatment delay may be required for severe symptoms (grade 3–4); resumption of therapy is possible in metabolically stable patients.

### 5.5. Pulmonary Toxicity

Pneumonitis occurs “frequently”, dyspnoea and cough “frequently” to “very frequently” depending on the ICI administered. Among all irAE, pneumonitis shows the highest mortality rate. Therefore, early diagnosis or differential diagnosis from “frequent” upper respiratory tract infections and pneumonia is important. The “very common” dysphonia is mainly VEGFR-TKI related and relatively less common under cabozantinib.

Pulmonary symptoms are frequently related to pulmonary metastases including disease progression. However, new or changing respiratory symptoms should always be thoroughly evaluated to exclude pulmonary toxicity. Symptomatic patients (e.g., upper respiratory tract infection, cough, shortness of breath, hypoxia) are evaluated by CT; high-resolution CT of the chest is favoured for differential diagnosis between pneumonia and immune-related pneumonitis. If pneumonitis is present, high-dose corticosteroid therapy should be initiated immediately. Pulmonary function and blood gases should be closely monitored. A chest X-ray should be performed at frequent intervals and, if necessary, infection excluded by bronchoscopy. This also allows safer initiation of immunosuppressive therapy, which in turn increases the risk of opportunistic infections [36]. If the differential diagnosis is uncertain, immunosuppressants and antibiotics should be administered simultaneously at an early stage. If pneumonitis is diagnosed as an (asymptomatic) incidental finding from imaging (grade 1), therapy can be continued but close monitoring is required. However, at the latest at grade 2 therapy must be delayed and in the case of recurrence permanently discontinued depending on the antibody or combination administered. The latter also applies to grade ≥ 3 irrespective of the ICI administered.

### 5.6. Renal Toxicity

Against first assumptions, “true” renal irAE are not common. Nevertheless, renal dysfunction (creatinine↑) has been observed “frequently” to “very frequently” with ICI therapy. Especially with combination therapies, also renal failure was “frequently” described. “Occasionally” nephritis occurs.

In the case of increased creatinine, other causes of renal insufficiency need to be ruled out (e.g., exsiccosis). For nephritis, proteinuria (urinalysis) may be indicative [37]. In case of doubt, a renal biopsy should be performed, which occasionally yields surprising findings (e.g., amyloidosis in our own patient cohort). For grade 2 nephritis, therapy should be interrupted and discontinued for grade 4. For grade 3, the recommended procedure depends on the ICI administered.

### 5.7. Cardiac Toxicity

Arterial hypertension is a “very common” VEGFR-TKI-associated adverse reaction and occurs in more than 50% of patients using VEGFR-TKI/ICI combinations. Depending on the administered medication, it is of higher grade (grade 3–4) in 12–25%. “Strict” blood pressure adjustment prior to therapy initiation, regular controls and, if necessary, adjustments of the antihypertensive therapy are mandatory (prescribe blood pressure monitor!).

Arrhythmias have been described “frequently”, especially with pembrolizumab ± axitinib. Nivolumab “occasionally” leads to tachycardia, in combination with ipilimumab “frequently”. “Rarely” to “occasionally”, potentially life-threatening myocarditis has been reported [38].

Cardiotoxicity may occur early after initiation of therapy. It may manifest nonspecifically (fatigue, hypotension) or directly as acute heart failure. Clinical symptoms as well as an increase in creatinine kinase require further evaluation (echocardiography, cardiac MRI, biopsy). Some patients could be successfully treated with high doses of corticosteroids, in other cases the outcome was fatal.

Treatment delay is recommended for grade 2. Treatment discontinuation is recommended for myocarditis ≥grade 3 or, for avelumab+axitinib for ≥grade 1 and confirmed diagnosis.

### 5.8. Neurological Toxicity

“Frequently” to “very frequently”, patients complain of headache, dizziness, peripheral neuropathies, lethargy and taste disturbances during ICI therapy. Differential diagnosis is sometimes difficult. In our own patient population, a facial nerve palsy was observed and classification as either irAE or “idiopathic” was hardly possible. Severe neurological toxicities are “rare”. These include encephalitis, myasthenia gravis and Guillain-Barré syndrome [39].

Brain metastases should be ruled out as the cause of symptoms by MRI and a neurologist should be involved early. Neurological irAE are treated with high-dose corticosteroids (prednisolone 1–2 mg/kg, p.o. or i.v.) and, if necessary, additional immunosuppressive measures. Permanent discontinuation of therapy is recommended from grade 3 at the latest.

## 6. Summary and Conclusions

Immunotherapy with ICI plays an important role in the treatment of patients with advanced or metastatic RCC. ICI frequently cause irAE which markedly differ from adverse reactions of other cancer drugs including VEGFR-TKIs and chemotherapeutics. Most frequently irAE involve the skin (rash, pruritus), gastrointestinal tract (colitis/diarrhoea), liver (hepatitis), endocrine system (thyroid disease), and lung (pneumonitis). However, any organ system can be affected. With the new VEGFR-TKI/ICI combination therapies in mRCC, the adverse reactions of both drugs appear to numerically add up. Common TKI-associated adverse reactions include diarrhoea, hypertension, fatigue, hypothyroidism, hand-foot syndrome, and gastrointestinal symptoms. These are managed by dose modification. In contrast, irAE lead to treatment delay or discontinuation and administration of corticosteroids or even more potent immunosuppressants. Differential diagnosis between irAE and TKI toxicity is sometimes difficult but crucial. When in doubt, TKI should be discontinued, and ICI infusion therapy delayed to safely establish a differential diagnosis. If both ICI and VEGFR-TKI have been interrupted, a sequential restart is recommended usually with the TKI being resumed first. Close multidisciplinary collaboration is essential for the safe use of ICI and early detection and management of toxicity.
